# A study of longitudinal mobile health data through fuzzy clustering methods for functional data: The case of allergic rhinoconjunctivitis in childhood

**DOI:** 10.1371/journal.pone.0242197

**Published:** 2020-11-17

**Authors:** Paolo Giordani, Serena Perna, Annamaria Bianchi, Antonio Pizzulli, Salvatore Tripodi, Paolo Maria Matricardi

**Affiliations:** 1 Department of Statistical Sciences, Sapienza University of Rome, Rome, Italy; 2 Department of Pediatric Pneumology and Immunology, Charitè Medical University of Berlin, Berlin, Germany; 3 Pediatric Unit, Mazzoni Hospital, Ascoli Piceno, Italy; 4 Practice of Pediatric Pneumology and Allergology, Berlin, Germany; 5 Pediatric Department and Pediatric Allergology Unit, Sandro Pertini Hospital, Rome, Italy; University of Defence, SERBIA

## Abstract

The use of mobile communication devices in health care is spreading worldwide. A huge amount of health data collected by these devices (mobile health data) is nowadays available. Mobile health data may allow for real-time monitoring of patients and delivering ad-hoc treatment recommendations. This paper aims at showing how this may be done by exploiting the potentialities of fuzzy clustering techniques. In fact, such techniques can be fruitfully applied to mobile health data in order to identify clusters of patients for diagnostic classification and cluster-specific therapies. However, since mobile health data are full of noise, fuzzy clustering methods cannot be directly applied to mobile health data. Such data must be denoised prior to analyzing them. When longitudinal mobile health data are available, functional data analysis represents a powerful tool for filtering out the noise in the data. Fuzzy clustering methods for functional data can then be used to determine groups of patients. In this work we develop a fuzzy clustering method, based on the concept of medoid, for functional data and we apply it to longitudinal mHealth data on daily symptoms and consumptions of anti-symptomatic drugs collected by two sets of patients in Berlin (Germany) and Ascoli Piceno (Italy) suffering from allergic rhinoconjunctivitis. The studies showed that clusters of patients with similar changes in symptoms were identified opening the possibility of precision medicine.

## Introduction

Mobile Health (mHealth) refers to the use of mobile communication devices in health care (see, e.g., [1]). In recent years, mHealth is rapidly growing. Nowadays, nearly every person possesses a mobile device and people carry their mobile device with them wherever they go. As observed by the World Health Organization (WHO), this holds for developed countries as well as for developing ones. Therefore, mobile devices might represent valuable tools in providing health care even to population in remote areas or with limited access to health care infrastructure by giving advices and taking recommendations for patients at low cost [2, 3].

The availability of mHealth data is exponentially increasing thanks to the impressive number of developed healthcare-related mobile apps [4]. These apps allow for effectively supporting disease prevention and management. In fact, for the first time, it is possible to monitor health conditions of patients, in particular, changes in health states, risk factors, daily behaviors and medication adherence. Healthcare-related apps usually acquire data by interactive questionnaires filled in by patients. When such questionnaires are repeated over a period of time or, in general, when mHealth data are collected on the same patients over time, data are said to be longitudinal, allowing for detecting developments or changes in the phenomenon under investigation. The analysis of such longitudinal mHealth data offers new opportunities to identify ad-hoc disease risks or symptom checkers and to generate customized diagnoses and treatment recommendations. The aim of this paper is to illustrate how this can be done by using two famous classes of statistical techniques, namely, Functional Data Analysis [5–7] and fuzzy cluster analysis [8, 9].

The need for Functional Data Analysis (FDA) naturally arises. In fact, a recognized drawback of (longitudinal) mHealth data is that they are full of noise [10]. For instance, in the case of interactive questionnaires, data quality may be poor due to a low confidence of patients with the app or to a limited accuracy in answering questions. Suitable techniques should be used to convert noisy mHealth data into valid and accurate information on the patients’ health status. Longitudinal mHealth data can be seen as realizations over time of continuous functions on a given domain. Since these measurements are observed with noise, the functions should be smooth in order to filter out the noise. This goal is achieved by means of FDA. FDA has often been applied in the biological and medical domains (see, for a review, [11]). Recent examples can be found in, e.g., [12–15].

Fuzzy cluster analysis is adopted in order to detect groups of patients with similar changes in the examined characteristics. Cluster-specific customized diagnoses and treatment recommendations can then be generated allowing for precision medicine. In practice, once noisy longitudinal mHealth data are converted into (smooth) functionals, these can be further analyzed by means of fuzzy clustering methods for functional data. The theory of fuzzy sets [16] represents an extension of the classical one where everything is a matter of degree. Formally, a unit (e.g. a patient) belongs to a fuzzy set with the so-called (fuzzy) membership degree. Such a degree *varies* from 0 (complete non-membership) or 1 (complete membership). This is obviously in contrast with the standard theory where a unit either belongs (membership = 1) or does not (membership = 0) to the set. Therefore, fuzzy set theory permits conclusions true to a certain extent admitting the selection of multiple options among a set of alternatives. In this respect, fuzzy set theory and its extensions offer more flexible tools for dealing with real-world complexity. Mathematical modelling based on fuzzy sets is widely applied in the medical domain as witnessed by the large number of papers available in the literature. For instance, fuzzy nonlinear systems can be considered [17–19]. A general model based on the novel concept of linear Diophantine fuzzy set is developed in [20]. Special kinds of fuzzy sets for the analysis of bipolar disorders are introduced in [21]. The so-called *m*-polar neutrosophic sets can be used for medical diagnosis [22]. Its applications to COVID-19 are described in [23, 24].

To further motivate the adoption of the fuzzy approach, we focus our attention to clustering. In standard (hard) clustering, units either belong or does not belong to the clusters. In fuzzy clustering, units can be assigned to the clusters with membership degrees in [0, 1]. Intuitively, units close to the cluster centers have membership degrees close to 1, while the membership degrees decrease as units are farther from the cluster centers. Finally, units on cluster boundaries, have similar membership degrees to more than one cluster highlighting cases of uncertain cluster membership. These cases are quite common in real life applications whenever there are units sharing the features of more than one cluster. They are not arbitrarily forced to fully belong to only one of such clusters, as it occurs in conventional clustering, but rather can be assigned to all the groups with certain membership degrees. The two most popular fuzzy clustering algorithms are the Fuzzy *k*-Means (F*k*M) algorithm [25] and the Fuzzy *k*-Medoids (F*k*Med) algorithm [26], representing fuzzy extensions of the classical *k*-Means (*k*M) [27] and *k*-medoids (*k*Med) [28] ones, respectively.

In this paper, the F*k*Med clustering algorithm for functional data is applied and introduced. The use of the *k*Med algorithm is motivated by its robustness properties [28] that make it more appropriate for mHealth data than the *k*M algorithm. In particular, the fuzzy *k*-medoids clustering algorithm for functional data is applied to longitudinal mHealth data concerning Allergic Rhinoconjunctivitis (AR). AR is an inflammatory disease of the nasal mucosa closely related to the aeroallergen exposure, such as pollen, house dust mites, etc. [29]. AR is one of the most common diseases among children and adults affecting over 300 million people, especially in industrialized countries [30]. Diagnosis and intervention trials depend on the severity of AR, that can be measured daily by patients answering questions related to the level of the perceived severity of symptoms and to the consumption of anti-symptomatic drugs. These answers allow for computing the so-called Symptom Medication Scores (SMS) in order to monitor the patients’ disease [31]. The present study is based on daily SMS referring to patients who collected daily symptoms and drug intakes by using the app AllergyMonitor (Technology Project & Software production, http://www.tpsproduction.com/en) developed within the project “Allergymonitor” [32], a multi-center project aimed at evaluating and validating the use of mobile Health Technology for allergy diagnostics.

## Materials and methods

### Functional data and clustering methods for functional data

FDA represents a set of statistical techniques used for analyzing experimental data, varying over a continuum, in the form of functions (see, e.g., [6]). If, for each unit, a collection of discrete observations over time is recorded, FDA allows for identifying and synthesizing the general trend of the discrete observed data. For the *i*-th unit (*i* = 1, …, *n*), this is done by converting the set of discrete values *y*_*ij*_ observed at times *t*_*ij*_ (*j* = 1, …, *v*_*i*_), where *v*_*i*_ denotes the number of discrete values for unit *i*, in a continuous smooth function *x*_*i*_(*t*), where *t* is in a continuum. Letting *ε*_*ij*_ be the error, we have

$$y_{ij}=x_{i}\left(t\right)+\varepsilon_{ij}.$$ (1)

The smoothing function *x*_*i*_(*t*) is created by using a basis function system, i.e., a set of *s* mathematically independent known functions, ϕ_*p*_, *p* = 1, …, *s*:

$$x_{i}\left(t\right)=\sum_{p}\;c_{ip}\phi_{p}\left(t\right),$$ (2)

where *c*_*ip*_ is the coefficient of ϕ_*p*_, *p* = 1, …, *s*. Different types of basis functions exist. A popular choice for non-periodic functional data is represented by spline functions. Their use is justified by the fact that a limited number of spline functions allow for remarkable flexibility in the data approximation. Splines are piecewise polynomials defined by dividing the observational time interval into *q* subintervals separated by usually equally-spaced points called *breakpoints* or *knots*, τ_*l*_, *l* = 1, …, *q–* 1. Note that, if the knots are not distinct, then the concepts of breakpoints and knots differ, but this occurs very rarely. In each sub-interval, the spline is a polynomial of order *m*. The order can be defined as the number of constants required to define it and is equal to the degree of the polynomial plus one. To improve flexibility in a spline, the number of breakpoints and the order of the polynomials can be increased at the cost of a more complex model with a high number of parameters.

In practice, the construction of the smoothing functions requires the definition of the functions ϕ_*p*_, *p* = 1, …, *s*. Once such functions are chosen, it remains to estimate the coefficients *c*_*ip*_, *p* = 1, …, *s*, for all the units. The most widespread choice for ϕ_*p*_ is represented by the B-spline basis system [33]. For the generic *i*-th unit, the smoothing function is obtained as follows. Let **Φ**_*i*_ be the matrix of order (ν_*i*_ × *s*) containing the values ϕ_*p*_(*t*_*ij*_) for unit *i*. Then, the estimation problem refers to the vector **c**_*i*_ = [*c*_*i*1_, …, *c*_*is*_]. Under the assumption that the errors are independent, identically distributed with zero mean and the same variance, the estimate of **c**_*i*_ is found by minimizing

$$SSE=\varepsilon_{i}{}^{T}\varepsilon_{i}={\left(y_{i}-\Phi_{i}c_{i}\right)}^{T}(y_{i}-\Phi_{i}c_{i}),$$ (3)

where **y**_*i*_ = [*y*_*i*1_, …, *y*_*ivi*_] and **ε**_*i*_ = [*ε*_*i*1_, …, *ε*_*ivi*_]. However, since the homoscedastic assumption is often unrealistic, a weight matrix is usually incorporated in the loss function in (3). The degree of smoothness of the estimated function depends on the number of basis functions *s*, leading to underfitting (low values of *s*) or overfitting (high values of *s*) problems. For this reason, a roughness penalty is usually added to (3) that explicitly defines the smoothness to be achieved. We have

$$PENSSE_{\lambda }=SSE+\lambda PEN_{m}\left(x\right),$$ (4)

where *PEN*_*m*_(*x*) = ∫[*D*^*m*^*x*_*i*_(*t*)]^2^d*t*, being *D*^*m*^*x*_*i*_(*t*) the *m*-th derivative of the function *x*_*i*_ at *t*. A common choice is *m* = 2, where the square of the second derivative of *x*_*i*_ at *t* defines the curvature degree of *x*_*i*_ at *t*. In (4), λ (>0) is a smoothing parameter that quantifies the emphasis of the roughness penalty in the loss function. The higher λ, the smoother the function *x*_*i*_. The smoothing parameter λ can be chosen by the well-known generalized cross-validation (GCV) measure [34]:

$$GCV=n\;SSE/{\left[n-df(\lambda )\right]}^{2},$$ (5)

where *df*(λ) denotes the degrees of freedom in the smoothing curve. The best choice of λ is associated with the minimum value of *GCV*.

By selecting *q*, *s* and λ, the functionals corresponding to the units can be determined. To identify functionals with similar features, clustering methods for functional data can be adopted. Such methods are recalled in the next section.

#### Clustering methods for functional data

Standard clustering methods assume to deal with a finite number of variables, i.e. to deal with a finite dimensional problem. As such, they are not adequate to cluster functional data lying on an infinite dimensional space. Functional clustering methods combine the functional representation of the observed data with a clustering algorithm in order to classify the units into groups. For this purpose, several suggestions have been proposed in the literature. For an overview, one may refer to [35] and references therein. A popular strategy consists in reducing the dimensionality of the problem by passing from an infinite dimensional space to a finite dimensional one. This goal can be achieved in terms of spline basis representations or functional principal component analysis [6]. The clustering method is then applied, in the former case, on the basis coefficients or, in the latter case, on the retained component scores. In this respect, the *k*M algorithm is often considered.

The first attempt to apply *k*M to the B-spline coefficients (*k*MFD) can be found in [35]. In *k*MFD, the prototypes (called centroids) are the average B-spline coefficients of the units assigned to the clusters. A few proposals suggest replacing the *k*M algorithm with the *k*Med one. These proposals, henceforth denoted by *k*MedFD, consist in applying *k*Med to the B-spline coefficients. In contrast with *k*M, the *k*Med prototypes are no longer fictitious entities, but a subset of the observed ones such that their dissimilarity to all the units in the cluster is minimal. Such prototypes are called medoids. This has two major advantages. The *k*Med prototypes are usually more robust to outliers than the *k*M ones. Moreover, the use of medoids simplify the cluster interpretation because *observed* entities can be used to describe the obtained clusters. For these reasons, we think that medoid-based algorithms are more appropriate for mHealth data than centroid-based ones. In the literature, several studies in the biological and medical domains involve the use of *k*MedFD [13, 36, 37]. All these applications are carried out following the classical approach to clustering. As far as we saw, studies adopting the fuzzy approach are not available. This paper aims at filling this gap. The Fuzzy *k*-Medoids algorithm for Functional Data (F*k*MedFD) is now introduced in detail by describing the two steps, labelled *fitting step* and *clustering step*, required to discover the functionals of the units and to identify clusters of functionals, respectively.

### Fuzzy *k*-medoids clustering method for functional data

The Fuzzy *k*-Medoids algorithm for Functional Data (F*k*MedFD) generalizes F*k*Med to functional data by means of two steps. In the first one (fitting step), the functionals are fitted to the observed data by means of B-splines. In the second step (clustering step), F*k*Med is applied to the B-spline coefficients obtained in the previous step. The two steps are related to each other because the optimal fitting and partitioning should be jointly determined, as we shall see in the application of Section 4.

#### Fitting step

In this step the functionals for all the units are built. In order to obtain comparable functional data, the same penalized B-spline functions should be used for all the units, setting the same number of knots, polynomial degree and smoothing parameter λ. For this purpose, a grid-search procedure can be implemented. For each combination of number of knots and polynomial degree, the smoothing parameter λ is determined in such a way to minimize

$$TGCV\left(q,m\right)=\sum_{i}GCV_{i},$$ (6)

where *GCV*_*i*_ is the *GCV* value for unit *i*, *i* = 1, …, *n*. Then, the optimal number of knots q* and the polynomial degree m* are

$$\left(q*,m*\right)=argmin_{q}{}_{,m}TGCV\left(q,m\right).$$ (7)

As we use the same number of knot and polynomial degree for all the *n* units, the same basis functions are used. It follows that, for each functional, the coefficients *c*_*ip*_, *p* = 1, …, *s* = q* + m* + 1, have the same meaning. Therefore, it is reasonable to use such coefficients for comparing the units in the clustering process. In fact, the obtained coefficients, stored in the matrix **C** of order (*n* × *s*), are used as input in the next step.

#### Clustering step

In the current step, the following constrained minimization problem is solved.

$$min_{U,H}\sum_{i}\sum_{l}u_{il}{}^{f}d^{2}\left(c_{i},h_{l}\right),$$ (8)

$$s.t.\;u_{il}\geq 0,\;i=1,\ldots ,n;l=1,\ldots ,k,$$ (9)

$$\sum_{l}u_{il}=1,\;i=1,\ldots ,n,$$ (10)

$$\left\{h_{l},\;l=1,\ldots ,k\right\}\subset \{c_{i},\;i=1,\ldots ,n\},$$ (11)

where *d* denotes the squared Euclidean distance and **U** is the membership degree matrix of order (*n* × *k*) with generic element *u*_*il*_ expressing the membership degree of unit *i* to cluster *l* (*i* = 1, …, *n*; *l* = 1, …, *k*). The elements of **U** belong to the interval [0, 1] and are such that their row-wise sum is equal to 1. Moreover, **H** is the medoid matrix of order (*k* × *s*). The vector **c**_*i*_ = (*c*_*i*1_, …, *c*_*is*_,), the *i*-th row of **C**, contains the coefficients for unit *i* and **h**_*l*_ = (*h*_*l*1_, …, *h*_*ls*_,), the *l*-th row of **H**, those for medoid *l*. Thus, a curve belongs to a cluster with a high membership degree when its coefficients have a small distance with respect to those of the cluster medoid. Of course, the medoids have membership degrees equal to 1 to the corresponding clusters (and equal to 0 to the remaining clusters). Finally, *f* (>1) is the so-called parameter of fuzziness. Its role is to tune the amount of fuzziness in the partition. High values of *f* imply *u*_*il*_ → 1/*k*, ∀*i*, *l*, whilst low values of *f* lead to *u*_*il*_ → {0, 1}, ∀*i*, *l*, i.e., the partition tends to be hard. Therefore, if a cluster should only comprise very similar units, *f* should be chosen larger [38]. It is important to note that several papers available in the literature present studies on the role and impact of the parameter of fuzziness on the obtained partition. Such works (e.g., [39–41]) usually concerns F*k*M. In medoid-based algorithms, it is recommended *f* ≤ 1.5 [26]. However, *f* cannot be objectively tuned. Its choice requires a lot of heuristic and a simple but useful recommendation is to perform several analyses setting different values of *f* and inspect whether and how the solutions differ.

The solution of the constrained minimization problem in (8)-(11) can be found by means of the method of Lagrange multipliers. The partial derivatives of the Lagrange function should be computed with respect to the parameters in order to find the stationary points of the Lagrange function. In doing so, it is convenient to split the optimization problem in two parts by treating **H** as a constant and minimizing with respect to **U** and vice-versa. In this way, the objective function is a convex function of **U** (and vice-versa). The updates of **U** and **H** should be repeated alternately until convergence.

The following iterative algorithm can be implemented.

**Step 0**: Set the number of clusters *k*, the parameter of fuzziness *f* and the convergence criterion ζ (>0, e.g. 10^−6^). Randomly select the membership matrix **U**^(*t*)^ with *t* = 0, provided that the constraints in (9) and (10) are fulfilled, where *t* denotes the iteration number.

**Step 1**: Considering **U**^(*t*)^ as fixed, update the medoid matrix **H**^(*t*+1)^. For the generic *l*-th row, we have

$$h_{l}{}^{\left(t+1\right)}=argmin_{i}\sum_{i’}u_{i’l}{}^{\left(t\right)f}d^{2}\left(c_{i},c_{i’}\right),\;l=1,\ldots ,k.$$ (12)

From (12) we observe that, for each cluster, the prototype, i.e., the medoid, is the observed unit such that the weighted sum of the distances between the unit involved and all the other ones with weights given by the membership degrees at the power of *f* is minimized.

**Step 2**: Considering **H**^(*t*+1)^ as fixed, update the membership degree matrix **U**^(*t*+1)^ as

$$u_{il}{}^{\left(t+1\right)}=d^{2}{\left(c_{i},h_{l}{}^{\left(t+1\right)}\right)}^{‐1/(f‐1)}/\sum_{l’}d^{2}{\left(c_{i},h_{l’}{}^{\left(t+1\right)}\right)}^{‐1/(f‐1)},i=1,\ldots ,n,l=1,\ldots ,k.$$ (13)

**Step 3**: Compute Δ = ||**U**^(*t*+1)^—**U**^(*t*+1)^||. If Δ > ζ, set *t*: = *t* + 1 and go to Step 1, otherwise consider the algorithm as converged.

It is not guaranteed that the above-described algorithm reaches the global minimum. To limit the chance of hitting local optima, more than one random start is recommended and the solution providing the lowest objective function value upon convergence should be selected.

**Remark**. Fuzzy *k*-means clustering methods for functional data.

By removing the constraint in (16), F*k*MedFD boils down to the Fuzzy *k*-means clustering methods for functional data (F*k*MFD). In this case, **H** is no longer the medoid matrix, but the prototype matrix. The solution of the F*k*MFD constrained minimization problem can be determined according to the iterative algorithm described in Section 3.2.2 provided that the update of **H** in (12) is replaced by

$$h_{l}{}^{\left(t+1\right)}=\sum_{i}u_{il}{}^{\left(t\right)f}h_{i}/\sum_{i}u_{il}{}^{\left(t\right)f},l=1,\ldots ,k.$$ (14)

Therefore, the centroids are now the weighted means of the units with weights corresponding to the membership degrees at the power of *f*.

The selection of the optimal number of clusters for F*k*MedFD is a complex issue. The optimal choice depends on the goals of the clustering process. Since clustering involves subjective judgements, the optimal number of clusters cannot be uniquely determined. However, in order to reach a decision, the use of fuzzy cluster validity criteria may help. A very common choice is the fuzzy silhouette index [42], which extends in a fuzzy setting the standard silhouette index [43]. Given a (hard) partition, we can compute the silhouette value for unit *i*:

s(i)=[b(i)−a(i)]/max[b(i),a(i)], (15)

where *a*(*i*) denotes the average dissimilarity between unit *i* and the other ones assigned to the same cluster and *b*(*i*) denotes the smallest average dissimilarity between unit *i* and the other ones assigned to the remaining clusters. The silhouette value ranges in [–1, 1]. If *s*(*i*) is close to 1, then unit *i* is well assigned to the cluster. Conversely, if *a*(*i*) approaches to –1, then the assignment of unit *i* is wrong. Values close to 0 mean that *i* shares the features of two clusters. The standard silhouette index is the average of the silhouette values:

$$S\left(k\right)=\sum_{i}s\left(i\right)/n.$$ (16)

The optimal number of clusters can be found in connection with the largest value of *S*(*k*).

In order to consider the fuzziness of the obtained partition, i.e., the membership degrees in **U**, the fuzzy silhouette (FS) index can be adopted:

$$FS\left(k\right)=\sum_{i}{\left(u_{ig}-u_{ig’}\right)}^{\gamma }s\left(i\right)/\sum_{i}{\left(u_{ig}-u_{ig’}\right)}^{\gamma },$$ (17)

where *u*_*ig*_ and *u*_*ig’*_ are the first and second largest elements of the *i*-th row of **U** and γ ≥ 0 is a weighting coefficient (usually γ = 1). The *FS*(*k*) index is a weighted mean of the silhouette values where the system of weights depends on the difference between the two largest membership degrees for every unit. In this way, the silhouette values of the units in the near vicinity of the cluster prototypes play a more relevant role if compared with those of the units located in overlapping areas. As for *S*(*k*), the optimal number of clusters can be found by maximizing the *FS*(*k*) index computed for different values of *k*. Nevertheless, in practice, we believe that this strategy may be too drastic. Specifically, we suggest inspecting not only the solution with the highest *FS*(*k*) value, but also those with values close to such a reference value. In fact, these alternative solutions may extract more relevant information.

### Data

F*k*MedFD was applied to mHealth data on children affected by AR. In particular, two studies, referring to two populations (from Ascoli, Italy, and Berlin, Germany) of patients suffering from AR, were considered. The study protocols were approved by the local responsible ethics committees, ethics committee of Charité Universitätsmedizin Berlin (approval number: EA2/004/13) for Berlin and Comitato Etico Asur (approval number: 46/CE-RMB, fascicolo n. 47/QQ, parere 383; date of approval 05/06/2009) for Ascoli Piceno. All parents or legal tutors provided written informed consent at the time of enrolment. The aim of these studies is to identify groups of children with similar levels of severity of symptoms allowing for (cluster-wise) tailored diagnoses and treatments.

#### Study population

mHealth data from two populations of children with seasonal allergic rhinitis and pollen sensitization in Berlin and Ascoli Piceno were analyzed. In Berlin, 31 children aged 5–18 years old were enrolled by a pediatric outpatient practice. All patients suffered from moderate-to-severe AR and grass pollen allergy. Exclusion criteria were the Immunoglobulin E sensitization to molds (e.g. Alternaria) and severe chronic diseases. The monitoring period started on May 14, 2013, and finished on June 12, 2013 (overall *v*_*i*_ = 30 days, ∀*i*). In Ascoli Piceno, 94 children aged 5–18 years old were enrolled by a pediatric outpatient clinic if they had mild or moderate-to-severe AR and grass pollen allergy with symptoms in May and/or June in at least one of the last two years. Exclusion criteria were the current or past administration of allergen immunotherapy for any pollen allergen and severe chronic diseases. The monitoring period started on May 13, 2011, and finished on June 21, 2011 (overall *v*_*i*_ = 40 days, ∀*i*). Both the populations of patients used the app AllergyMonitor. Its use was explained to patients during the medical visits. Patients could enter daily data on the app during the same day or not later than the day after. For each patient, the functional was built according to a finite number of SMS values during a reference time.

## Results and discussion

The results of the two above-mentioned studies involving the application of the F*k*MedFD algorithm are reported. For comparative purposes, results obtained by using potential competitors are also given. All the analysis was implemented by the open source statistical software R [44]. In particular the packages fda [45] for FDA and fclust [46] for fuzzy cluster analysis were used.

### Preliminary analyses

#### CSMS values

The first step of the analysis was the computation of daily SMS values on the basis of the daily mHealth data recorded by AllergyMonitor. In the literature, several SMS indexes have been developed to measure the severity of AR [19, 47]. With the aim to make clinical studies comparable, a task force of the European Academy of Allergy and Clinical Immunology recommended the use of the so-called Combined Symptom and Medication Scores (CSMS) index [48, 49]. The CSMS index represents a simple tool balancing both the symptoms and the need for antiallergic medication in an equally weighted manner. Symptoms are measured by the Average Rhinoconjunctivitis Total Symptom Score (ARTSS). For each patient, the daily ARTSS value ranges in the interval [0, 3] and is equal to the average values of the symptoms for six clinical features, i.e., sneezing, rhinorrhea, pruritus and nasal congestion (nasal symptoms), ocular pruritus and lacrimation (ocular symptoms). The consumption of anti-symptomatic drugs is evaluated by the Rescue Medication Score (RMS) that measures the category of anti-symptomatic drugs that the patient is taking. Increasing scores from 0 to 3 are assigned to different categories of drugs, taking into account their intensity (0 = no medication, 1 = antihistamine, 2 = nasal corticosteroid, 3 = oral corticosteroid). For a given day the RMS is equal to the highest score recorded by the patient. CSMS is the sum of ARTSS and RMS and thus takes values in the interval [0, 6].

#### Pollen data

Pollen data were also observed. In particular, for Ascoli Piceno, the counts of three pollens were recorded allowing to relate the patients’ symptoms to a specific pollen. Such a study was not carried out for Berlin, because only one pollen was recorded. Pollen data for Berlin (Gramineae) were provided by the Institute of Meteorology of the Freie Universität Berlin and those for Ascoli Piceno (Gramineae, Olea, Urticaceae, Cupressaceae) by the aerobiology center of the Agenzia Regionale per la Protezione Ambientale delle Marche, located in Castel di Lama (Ascoli Piceno). Pollen counts were expressed as grains/m^3^. The pollen curve of Berlin was characterized by a phase of absence or paucity of pollens in the atmosphere during May and the appearance of moderate concentration in June. The pollen season of Ascoli Piceno was very prosperous, with peaks of over 400 grains/m^3^ for the Olea and almost 200 grains/m^3^ for the Gramineae. Cypress pollens reached concentrations of negligible clinical relevance and, hence, were not considered in the study.

For comparative purposes, daily CSMS and pollen values were normalized in such a way to vary in the interval [0, 1]. Specifically, for the CSMS index, values were divided by 6, whilst, for each pollen, values were divided by the maximum observed level during the reference time.

#### Missing data

Several missing data were observed. In fact, some patients did not access the app daily or some others partially filled in the form. We assumed that, in a given day, no available information on symptoms and drug intakes denoted missingness. Instead, if in a given day only symptoms were recorded, we assumed that the patient did not take any medication, i.e., RMS = 0.

We imputed missing values as in [50]. Specifically, for the generic *i*-th patient, if the first available value was *y*_*it’*_, with *t*’ > 1, the imputed value *y*_*it*_^I^ was set equal to *y*_*it’*_, ∀*t* = 1, …, *t*’–1; if the last available value was *y*_*it’*_, then *y*_*it*_^I^ *= y*_*it’*_, ∀*t* = *t*’, …, *v*_*i*_. Finally, intermediate missing data were imputed by interpolation. For instance, if the values {*y*_*it’*+1_, …, *y*_*it’+w*-1_} were missing, then *y*_*it’+t”*_^I^ = *y*_*it’*_ + (*y*_*it’+w*_*−y*_*it’*_)*t*”/*w*, ∀*t*” = 1, …, *w*–1. It is suggested imputing missing data for patients that did not exceed 25% of missing values [50]. In our case, this cut-off had dramatic consequences especially in the Berlin population leading to a subpopulation of only 21 patients (10 out of 31 patients excluded). For this reason, we decided to consider a milder cut-off equal to 37.5% such that *n* = 26 patients were analyzed in Berlin. Regarding Ascoli Piceno, 22 patients with more than 37.5% of missing values were excluded from the analysis. Moreover, two patients were managed as outliers and thus excluded. The first one daily recorded no symptoms and no drug intakes. The second one always recorded the same daily values. This led to *n* = 71 patients included in the analysis. The extent to which the results were affected by the cut-off was investigated by a small sensitivity analysis considering an extremely mild and a severe cut-off equal to 50% and 25% respectively.

### Statistical analysis

In both the studies for each patient, the daily CSMS values were considered to build the corresponding functional. Then, we used the coefficients of these functionals as input for F*k*MedFD in order to discover groups of patients. The functionals were built by varying the number of equidistant B-spline knots (5, 10, 15 and 20, labelled, respectively, 6-days, 3-days, 2-days and 1.5-days for Berlin and 8-days, 4-days, 3-days and 2-days for Ascoli Piceno) and the order of the B-spline function (polynomial degree equal to 3 and 5, respectively, *m* = 4 and 6). The smoothing parameter λ was equal to 10^*g*^, where *g* took values in the interval [–30, 20] with increasing step equal to 0.05 [51]. The optimal parameters were selected according to (7). However, when the differences in the optimal *TGCV* values were negligible, for the sake of parsimony the coefficients of the simpler model were used as input in F*k*MedFD. Concerning F*k*MedFD, the optimal number of clusters was chosen by inspecting the solutions with the highest value of the fuzzy silhouette index varying *k* from 2 to 7 (for Berlin) or 10 (for Ascoli) and setting *f* = 1.5. Once *k* was selected, we checked whether different partitions were obtained by varying *f*. In both the studies we found that the same groups of patients were identified but, obviously, the larger *f*, the fuzzier the membership degrees.

#### Berlin

The functionals were constructed by considering cubic B-splines and 15 knots. This choice did not lead to the minimum *TGCV*. Nevertheless, it represented the best compromise between computational feasibility and feature reproduction. In fact, by considering the case 1.5-days, the *TGCV* value was slightly lower than that obtained in the case 3-days, but the number of parameters noticeably increased, without any advantage in the obtained partition, as we shall see later. The details on the performance of the various models in terms of *TGCV* are reported in Table 1.

**Table 1:** Model selection for the functional data: Best values of *TGCV* for different choices of *m*, *q* and λ.

Functional model	*m*	*q*	λ	*TGCV*
B-splines 6-days	4	5	35.48	0.0902
B-splines 6-days	6	5	35.48	0.0899
B-splines 3-days	4	10	25.12	0.0891
B-splines 3-days	6	10	19.95	0.0887
B-splines 2-days	4	15	1.26	0.0861
B-splines 2-days	6	15	1.26	0.0859
B-splines 1.5-days	4	20	1.26	0.0860
B-splines 1.5-days	6	20	1.26	0.0859

In order to select the number of clusters, we found that the highest *FS*(*k*) value was registered when *k* = 2 (0.64) whilst, when *k* = 3, it was equal to 0.59. The values sensibly decreased when *k* = 4 and 5 (0.50 and 0.40, respectively) and fell to about 0 for higher values of *k*. For all of these reasons, we concentrated our attention to the solutions with *k* = 2 or 3. When *k* = 3, the sizes of Clusters 1, 2 and 3 were 12, 9 and 5, respectively, by considering the maximum membership degree. Note that, in the hard-clustering sense, i.e., a unit is assigned to a cluster when the maximum membership degree is higher than 0.50, three patients (two from Cluster 1 and one from Cluster 3) had unclear assignments. This occurred because these patients shared the characteristics of more than one cluster.

A deeper analysis of the three clusters highlighted that the membership degrees of the patients assigned to Clusters 2 and 3 were rather fuzzy. In fact, except for the medoids, all the patients had membership degrees lower than 0.70. This suggested that a too fragmented grouping of the patients was found and that these two clusters should probably be joined. We thus investigated the solution with *k* = 2 clusters. Clusters 1 and 2 had sizes equal to 12 and 14, respectively. Cluster 2 was mainly composed by the patients belonging to Clusters 2 and 3 found setting *k* = 3. This confirmed that the solution with *k* = 2 clusters should be the preferred one. Moreover, the membership degrees were high (≥ 0.90) for 19 patients (9 for Cluster 1 and 10 for Cluster 2). Thus, two well separated clusters seemed to exist.

**Fig 1:**
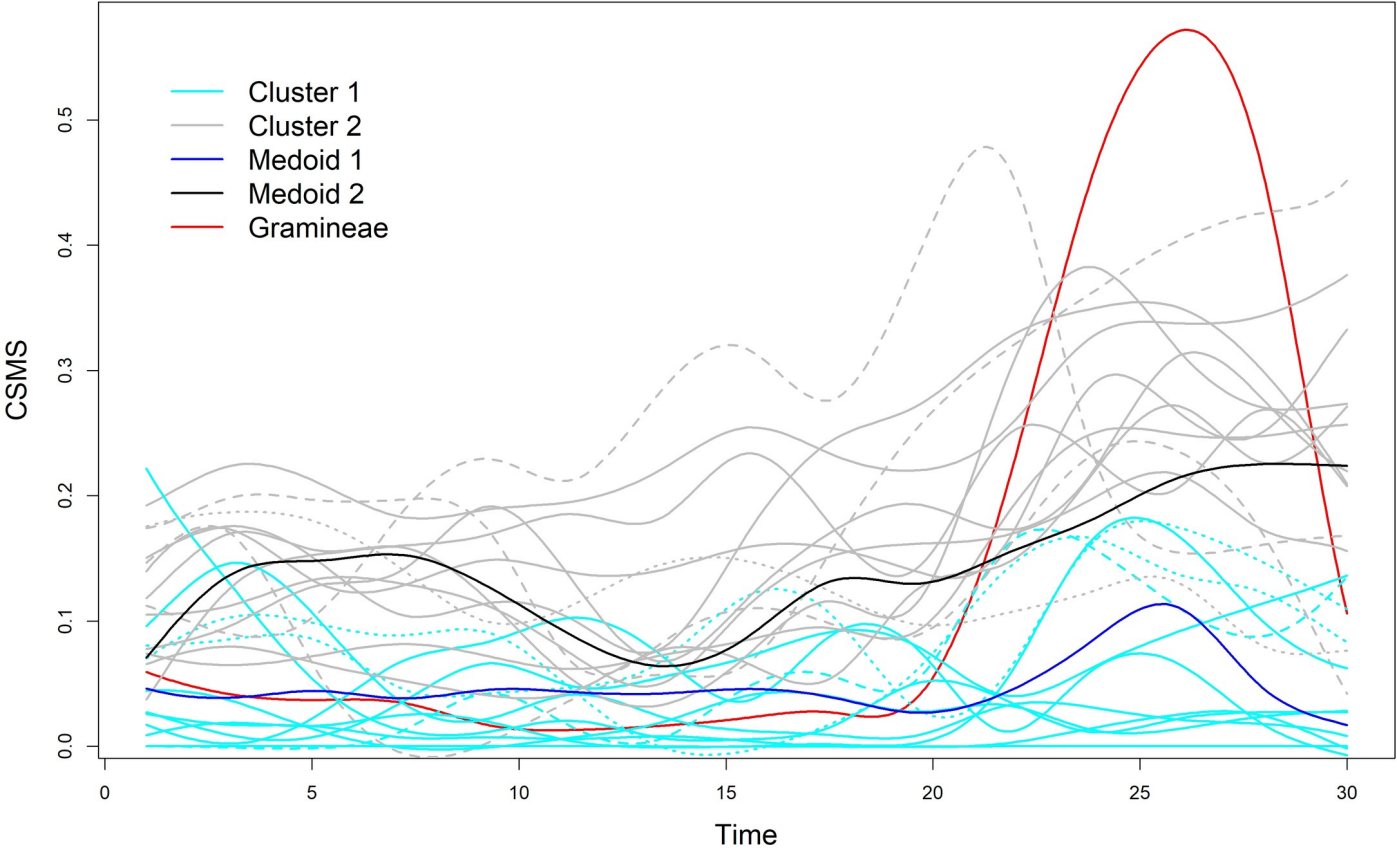
Plot of the F*k*MedFD solution and of the pollen (red functional). Cyan and grey functionals identify patients assigned to Cluster 1 (medoid in blue) and Cluster 2 (medoid in black), respectively. Solid, dashed and dotted functionals denote membership degrees higher than 0.90, between 0.70 and 0.90 and between 0.50 and 0.70, respectively.

To further inspect the obtained clusters, we plotted the functionals distinguishing the cluster memberships and the medoids in Fig 1. Note that the figure also contains the functional for the Gramineae, built by using *m* = 3 and *q* = 15, in order to interpret the clusters in terms of the pollen. Moreover, the characteristics of the clusters were analyzed by considering demographic and clinical information (Table 2), the severity of symptoms and the intake of anti-symptomatic drugs (Table 3). In particular, Table 3 contains the average scores of three alternative indexes for AR during the reference time and the last two weeks (i.e., during the pollen peak). Two of them were RMS and ARTSS, used to build CSMS. By considering RMS and ARTSS separately, the aim was to assess whether high CSMS values depended on severe symptoms, high levels of anti-symptomatic drug intakes or both. The third index, called ACS (see, e.g., [50]), considers not only nasal and ocular symptoms (as is for ARTSS), but also bronchial symptoms (cough, wheezing and dyspnea). For this reason, larger values of ACS compared with those of ARTSS represent a proxy of asthma diseases and, therefore, help to discover patients with severe disease.

**Table 2:** Demographic and clinical information grouped by cluster.

	Cluster 1 (*n* = 12)		Cluster 2 (*n* = 14)		*p*-value
Males (*n*, %)	6	50.0	9	64.3	0.736
Age (years) (mean, SD)	13.6	2.6	12.3	2.8	0.233
Nationalilty (*n*, %)					
German	10	83.3	8	57.1	0.216
Others	2	16.7	6	42.9	
Atopic sensitization (*n*, %)					
Birch pollen	8	72.7	9	69.2	1.000
Dermatophagoides spp.	4	36.4	3	23.1	0.659
Others	8	72.7	9	69.2	1.000
Desensitization (*n*, %)	10	90.9	11	78.6	0.604
Asthma (*n*, %)	6	54.5	9	64.3	0.697
Atopic dermatitis (*n*, %)	2	18.2	0	0.0	0.183
Duration of allergy (years) (mean, SD)	6.2	1.3	4.3	2.3	0.043

Note: Quantitative data are summarized as mean and standard deviation (SD) and categorical data as frequency (*n*) and percentage (%). The *p*-values are computed by T-test, when conditions were met, or Mann-Whitney U-Test for quantitative data and Chi square test, when conditions were met, or Fisher exact test for categorical data.

**Table 3:** Severity of symptoms and intake of anti-symptomatic drugs grouped by cluster.

	Cluster 1 (*n*_1_ = 12)		Cluster 2 (*n*_2_ = 14)		*p*-value
Average RMS					
total period-30 days (mean, SD)	0.01	0.03	0.19	0.16	0.006
last 14 days (pollen peak) (mean, SD)	0.03	0.07	0.43	0.37	0.006
Average ARTSS					
total period-30 days (mean, SD)	0.26	0.16	0.82	0.27	<0.001
last 14 days (pollen peak) (mean, SD)	0.30	0.25	0.89	0.36	<0.001
Average ACS					
total period-30 days (mean, SD)	1.93	1.22	6.15	1.95	<0.001
last 14 days (pollen peak) (mean, SD)	2.17	1.93	6.86	2.83	<0.001

Note: Data are summarized as mean and standard deviation (SD). The *p*-values are computed by T-test, when conditions were met, or Mann-Whitney U-Test.

By inspecting Fig 1 we can see that the patients assigned to Cluster 2 were affected by more severe AR, if compared with those belonging to Cluster 1. This is easily visible for the curves corresponding to the two medoids. During the first half of the reference time, the pollen concentrations were very low and, except for a few cases, the symptoms of the patients of Cluster 2 were slightly more intense than those of Cluster 1. During the second half of the reference time, the values of almost all the functionals increased. This means that, during the pollen-peak days, all the patients suffered from more severe symptoms. Such a result can be seen by observing the functional of the medoid of Cluster 1 (the one in blue). In fact, the maximum value of such a curve occurred together with the maximum value of the pollen curve. During the pollen-peak days, the differences between the clusters were more evident. It follows that the pollen especially affected the patients belonging to Cluster 2. A couple of patients belonging to Cluster 1, in particular those having the highest values of the curves around day 25, suffered from slightly more severe symptoms. Such patients, denoted by dotted functionals, were well captured by the clustering method because were characterized by the lowest membership degrees to Cluster 1 (0.66 and 0.69). The opposite comment holds for one patient assigned to Cluster 2 with the lowest membership degree (0.59). Her/his dotted curve allowed us to identify a patient characterized by medium symptoms approximately between those of the two medoids. The remaining patients were strongly assigned to the clusters, i.e., with membership degrees higher than 0.90 except for a few patients (one from Cluster 1 and three from Cluster 2) with membership degrees belonging to the interval [0.70, 0.90). These patients can be recognized by dashed functionals quite far from the corresponding medoid and very far from the medoid of the other cluster. For instance, these are clearly visible on the top of the figure, hence referring to patients with extremely severe symptoms.

By looking at the demographic and clinical information reported in Table 2, we can see that Cluster 1 was composed by a larger percentage of German and slightly older patients. More cases of desensitization ad atopic dermatitis were registered for Cluster 1, whilst Asthma was observed for the patients belonging to Cluster 2 with a higher percentage. However, such variables were not significantly different between clusters. The only clinical information playing a significant role in distinguishing the two clusters seemed to be the duration of allergy that was longer for Cluster 1.

The results of Table 3 showed that the scores of RMS, ARTSS and ACS increased with respect to time. This means that higher values of CSMS were related to high values of both RMS and ARTSS. In particular, the registered values were higher for patients assigned to Cluster 2 with respect to those assigned to Cluster 1. This especially holds during the pollen-peak days. With respect to ACS, we observed large values for Cluster 2, particularly during the pollen-peak days. We can thus conclude that the patients assigned to Cluster 2 suffered from asthma symptoms in a more pronounced way in comparison with those belonging to Cluster 1.

Overall, the partition classified the patients into two clusters according to the severity of AR. Cluster 1 was interpreted as ‘‘mild symptoms” and Cluster 2 as ‘‘severe symptoms”. In the Berlin data, the severity of AR was inversely related to the duration of allergy. Therefore, the patients having a longer history of AR had milder symptoms.

The assessment of the stability of the obtained partition was carried out by means of a sensitivity analysis varying the chosen parameters. First of all, we noted that different values of the parameter of fuzziness *f* led to the same partition. The only differences were in the membership degrees. Moreover, we investigated whether the solution differed by building the functional data by setting *m* = 6 and *q* = 20. This was the most complex model leading to the lowest *TGCV* value. We found that the same clusters were discovered by using *k* = 2 and *f* = 1.5. Specifically, the same medoids described the clusters and the membership degrees were very similar between the two solutions. The largest difference between corresponding membership degrees was 0.03. Furthermore, we checked how the cut-off for the missing values affected the results. We observed that the use of the cut-offs equal to 50% and 25%, respectively, did not modify the obtained partition.

Finally, we compared the previously described partition with those obtained by applying alternative clustering methods, summarized in Table 4. The comparison was not only limited to clustering methods according to the hard or fuzzy approaches. In fact, we also considered probabilistic clustering methods where the posterior probabilities (taking values in [0, 1]) play the role of the fuzzy membership degrees. Probabilistic and fuzzy clustering methods produce *soft* partitions to distinguish them from clustering methods built according to the classical approach producing *hard* partitions. We were interested in the solutions with *k* = 2 clusters. These solutions were found by R functions run by using default options and setting *f* = 1.5 for the fuzzy clustering methods. Some clustering methods are tailored for functional data. For comparative purposes, the same functional data, i.e., setting the same values of *m*, *q* and λ as for F*k*MedFD, were used as input in the R functions. On the contrary, the clustering methods for standard data were applied to the raw (observed) data. Since raw data were assumed to be noisy, we expected different (and worse) results if compared with those from methods for functional data.

**Table 4:** Alternative clustering methods used for comparison purposes.

Method	Use of functional data	Hard/Soft partition	Centroid/Medoid based	R function
funFEM [52]	Yes	Soft	Centroid	funFEM [53]
funHDDC [54]	Yes	Soft	Centroid	funHDDC [55]
*k*MFD	Yes	Hard	Centroid	kmeans [44]
*k*MedFD	Yes	Hard	Medoid	pam [56]
F*k*MFD	Yes	Soft	Centroid	FKM [46]
*k*M	No	Hard	Centroid	kmeans [44]
*k*Med	No	Hard	Medoid	pam [56]
F*k*M	No	Soft	Centroid	FKM [46]
F*k*Med	No	Soft	Medoid	FKMed [46]

**Fig 2:**
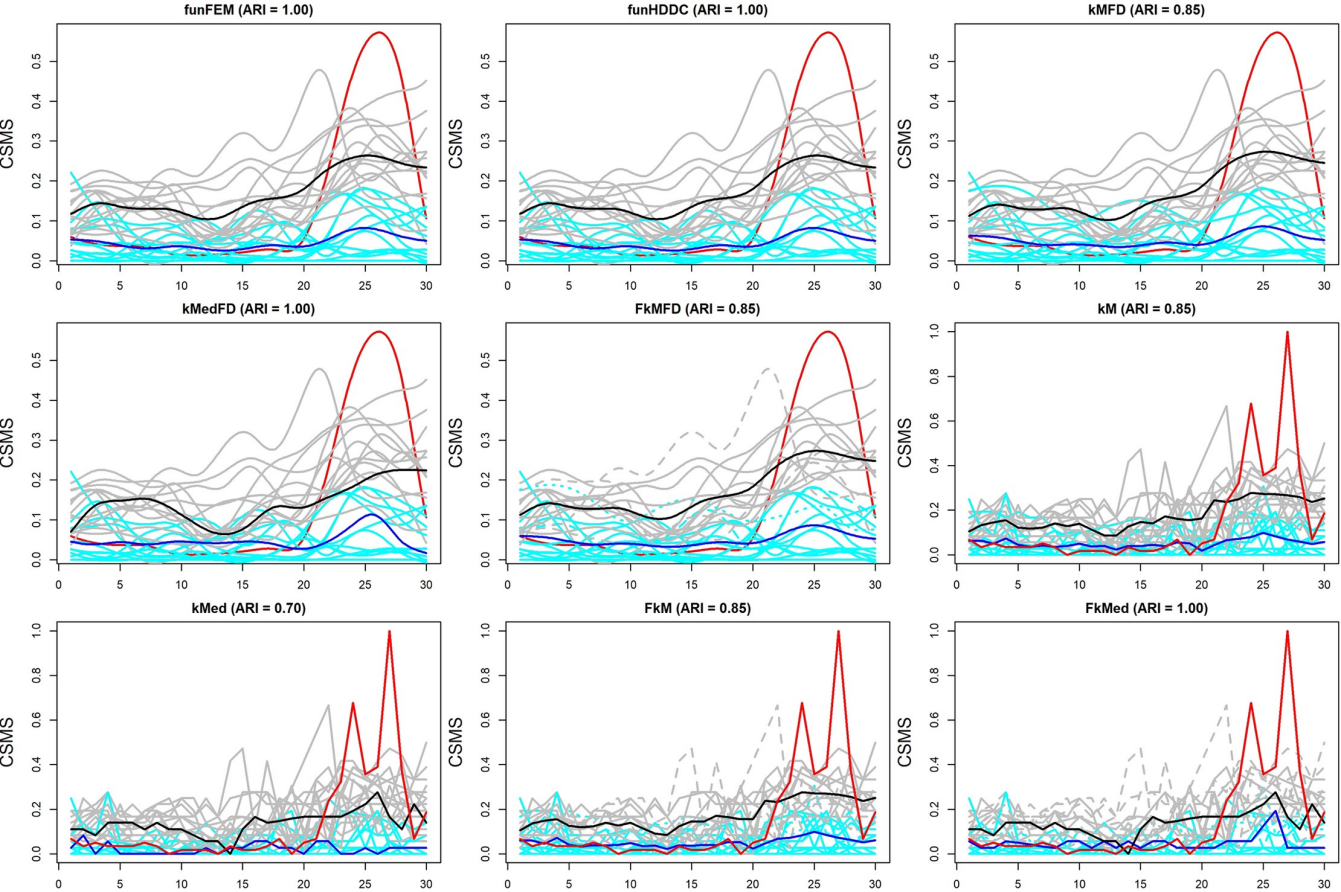
Plot of the solutions of the methods reported in Table 4 and of the pollen (red functional). Cyan and grey functionals identify patients assigned to Cluster 1 (medoid in blue) and Cluster 2 (medoid in black), respectively. Solid, dashed and dotted functionals denote membership degrees higher than 0.90, between 0.70 and 0.90 and between 0.50 and 0.70, respectively.

The results of the comparison are summarized in Fig 2 containing the plots of the obtained partitions and the corresponding Adjusted Rand Index (ARI) [57] values computed with respect to the F*k*MedFD solution. As is well-known, ARI is a measure of the similarity between two partitions such that ARI = 1 means perfect agreement. Although variants of ARI for soft partitions exist [58], we chose the traditional ARI measure because we were interested in comparing hard and soft partitions. To this purpose, soft partitions were converted into hard ones in the hard clustering sense.

By inspecting Fig 2, we can observe a general agreement among the solutions. In particular, four methods (funFEM, funHDDC, *k*MedFD and F*k*Med) produced the same partition as for F*k*MedFD. Nevertheless, some differences emerged. Specifically, funFEM and funHDDC assigned the patients to the clusters with posterior probabilities essentially equal to 1 even if, as already observed, some functionals shared the features of both the clusters and therefore more uncertainty in the cluster assignment would be desirable. On the contrary, F*k*Med led to quite low membership degrees for a large number of patients, in particular those assigned to Cluster 2. This might be explained by the use of raw data containing noise that implies too high distances with respect to the medoids. By comparing the F*k*MedFD partition with those provided by the remaining five methods we observed only one or two patients assigned to a different cluster. Such patients had rather uncertain memberships because their symptoms and drug intakes were in between the medoids or centroids of the two clusters.

#### Ascoli Piceno

The results of the analysis for the patients from Ascoli Piceno are reported in this section. There were at least two relevant differences with respect to the Berlin study, namely, a larger number of patients (*n* = 71 by using the cut-off for missing data equal to 37.5%) and the availability of data on three pollen concentrations (Gramineae, Olea, Urticaceae). The latter information was used in order to assess whether the clusters could be interpreted by the pollen counts and to relate the patient allergy to a specific pollen. The most compelling models are summarized in Table 5 where the number of knots, the degree of the polynomials, the penalization coefficient and the *TGCV* value are reported.

**Table 5:** Model selection for the functional data: Best values of *TGCV* for different choices of *m*, *q* and λ.

Functional model	*m*	*q*	λ	*TGCV*
B-splines 8-daily	4	5	14.13	0.6047
B-splines 8-daily	6	5	1.58	0.5778
B-splines 4-daily	4	10	3.98	0.5288
B-splines 4-daily	6	10	3.98	0.5239
B-splines 3-daily	4	15	2.82	0.5100
B-splines 3-daily	6	15	2.82	0.5089
B-splines 2-daily	4	20	1.78	0.5015
B-splines 2-daily	6	20	2.00	0.5011

We can see that the minimum *TGCV* value was found when *m* = 6 and *q* = 20. Nevertheless, this model was the most complex one and therefore we decided to consider the more parsimonious one with *m* = 4 and *q* = 20.

The F*k*MedFD clustering algorithm was then applied by varying the number of clusters. We got decreasing scores of the *FS*(*k*) index for increasing values of *k* passing from *FS*(*k*) = 0.67 when *k* = 2 to *FS*(*k*) = 0.38 when *k* = 10. This result suggested that a limited number of groups was needed to cluster the patients. Nevertheless, preliminary studies showed that the solution with only *k* = 2 clusters oversimplified the patients’ taxonomy. For this reason, we preferred the partition obtained setting *k* = 3 for which *FS*(*k*) = 0.58.

By considering the maximum membership degrees, the sizes of Clusters 1, 2 and 3 were 27, 23 and 21, respectively. However, the fuzzy approach to clustering allowed for identifying six patients not clearly assigned, i.e., with highest membership degrees lower than 0.50. All of them had membership degrees slightly lower than 0.50 to two clusters. Hence, these patients shared the features of the two involved clusters. Clusters 1 and 2 closely resembled the ones obtained by setting *k* = 2. Specifically, Cluster 1 was composed by patients with severe symptoms during the entire reference time and, hence, labelled ‘‘severe symptoms”. On the contrary, Cluster 2 was characterized by ‘‘mild symptoms”. Therefore, such two clusters seemed to distinguish the patients with respect to high and low values of CSMS. More interestingly, Cluster 3 discovered some patients suffering from severe symptoms during the first half of the reference time and from mild symptoms during the second half.

**Fig 3:**
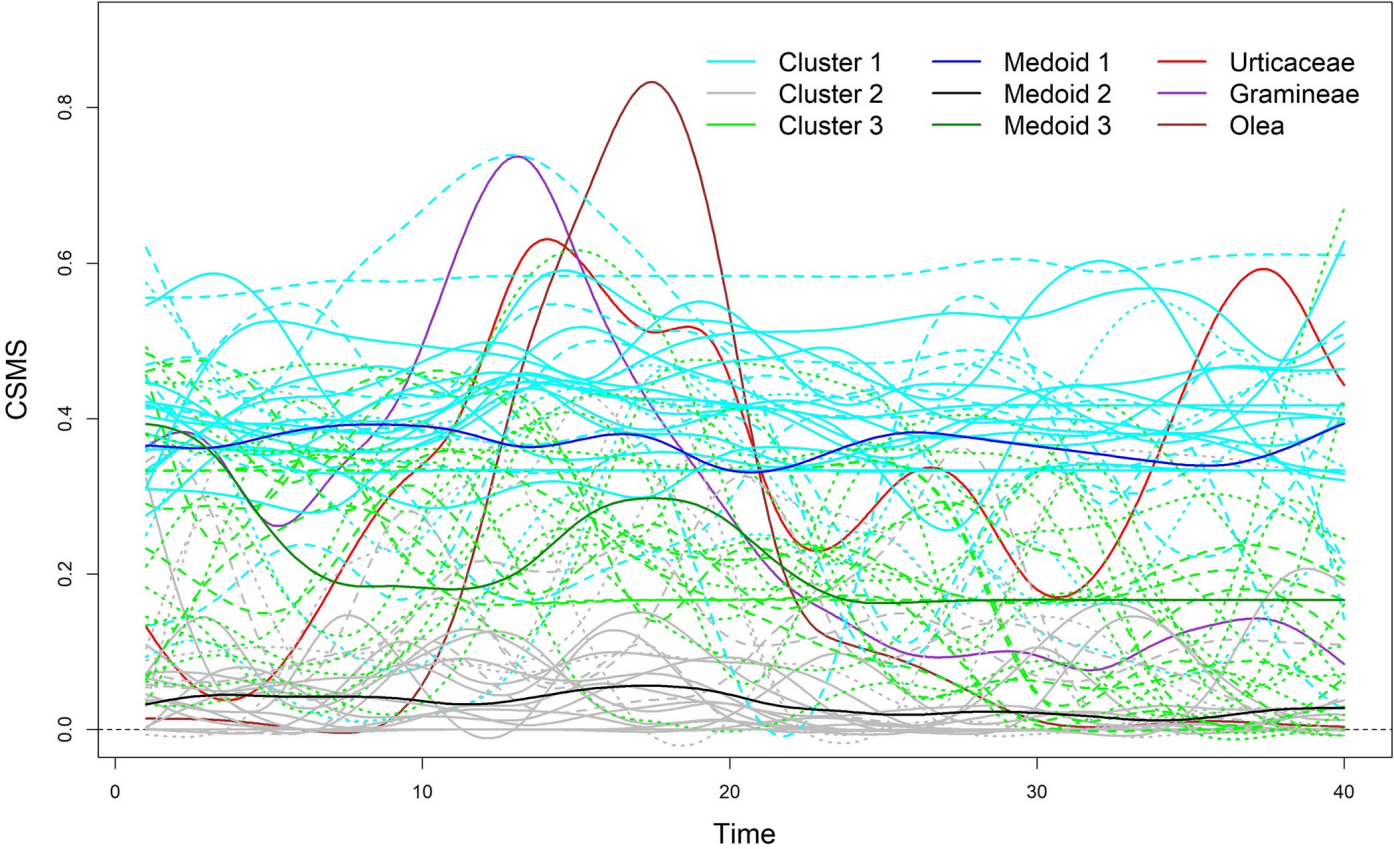
Plot of the F*k*MedFD solution and of the pollens (red, violet and brown functionals). Cyan, grey and green functionals identify patients assigned to Cluster 1 (medoid in blue), Cluster 2 (medoid in black) and Cluster 3 (medoid in dark green), respectively. Solid, dashed and dotted functionals denote membership degrees higher than 0.90, between 0.70 and 0.90 and between 0.50 and 0.70, respectively.

All of these findings can be observed by looking at Fig 3, where the functionals of the patients grouped by cluster and of the pollens are displayed. From the figure, we can also see that the peaks of Olea and Gramineae occurred between Day 10 and Day 20. During the same days, the peaks of several functionals for patients assigned to Cluster 3 (for instance Medoid 3) are visible. This stimulated us in order to assess whether a relationship between the patients of Cluster 3 and the pollens Olea and Gramineae existed. This point will be further discussed below.

To aid the cluster interpretation we considered some demographic and clinical information (Table 6) and the severity of symptoms and the intake of anti-symptomatic drugs by means of RMS, ARTSS and ACS (Table 7). From Table 6, we found that the clusters were very similar in terms of the demographic features of the patients. With respect to the clinical characteristics, we observed lower percentages of the allergic comorbidities for the patients of Cluster 2 with respect to those assigned to the other clusters. All the indexes reported in Table 7 were significantly different among the clusters.

**Table 6:** Demographic and clinical information grouped by cluster.

	Cluster 1 (*n*_1_ = 27)		Cluster 2 (*n*_2_ = 23)		Cluster 3 (*n*_3_ = 21)		*p*-value	
Males (*n*, %)	20	74.1	16	69.6	15	71.4	0.938
Age (years) (mean, SD)	10.4	3.1	10.0	3.7	9.3	3.1	0.503
Nationality (*n*, %)							
Italian	26	96.3	22	95.7	21	100.0	0.859
Others	1	3.7	1	4.3	0	0.0	
Allergic comorbidities (*n*, %)							
Asthma	7	25.9	5	21.7	8	38.1	0.459
Oral allergy syndrome	3	11.1	3	13.0	3	14.3	1.000
Anaphylaxis	3	11.1	2	8.7	2	9.5	1.000
Urticaria and/or angioedema	3	11.1	4	17.4	0	0.0	0.159
Atopic dermatitis	9	33.3	6	26.1	10	47.6	0.330
Gastrointestinal symptoms	1	3.7	0	0.0	0	0.0	1.000
Atopic reactivity (mean, SD)							
Overall SPT reactivity to pollens (mm)	45.3	33.1	46.6	32.4	46.3	24.1	0.912
Number of positive SPT	7.2	5.4	7.7	5.4	7.8	4.4	0.792
Duration of allergy (years) (mean, SD)	3.7	2.0	3.7	2.3	3.0	1.3	0.556

Note: Quantitative data are summarized as mean and standard deviation (SD) and categorical data as frequency (*n*) and percentage (%). The *p*-values are computed by one-way ANOVA, when conditions were met, or Kruskal-Wallis test for quantitative data and Chi square test, when conditions were met, or Fisher exact test for categorical data.

**Table 7:** Severity of symptoms and intake of anti-symptomatic drugs grouped by cluster.

	Cluster 1 (*n*_1_ = 27)		Cluster 2 (*n*_2_ = 23)		Cluster 3 (*n*_3_ = 21)		*p*-value
Average RMS total period-40 days (mean, SD)	1.75	0.33	0.14	0.20	0.90	0.46	<0.001
Average ARTSS total period-40 days (mean, SD)	0.57	0.35	0.26	0.21	0.46	0.31	0.001
Average ACS total period-40 days (mean, SD)	4.51	3.09	1.81	1.35	3.62	2.29	<0.001

Note: Data are summarized as mean and standard deviation (SD). The *p*-values are computed by one-way ANOVA, when conditions were met, or Kruskal-Wallis test.

Consistently with the cluster interpretation, the highest and lowest average values of RMS, ARTSS and ACS were registered for Clusters 1 and 2, respectively. Note that Table 6 does not contain scores on the indexes for the pollen-peaks because the three pollen-peaks occurred in different occasions.

In order to further interpret the clusters in terms of the pollens, we developed the so-called Allergic Rhinoconjunctivitis-pollen (*ARp*) index, aiming at assessing, for each patient, the relationship between the CSMS functionals and the pollen ones. The index takes scores in the interval [0, 1] and expresses the extent to which a patient is related to a pollen curve. From a clinical point of view, this index may have relevant consequences in helping doctors to address a patient to a specific immunotherapy.

The *ARp* index was built as follows. For each patient *i* (*i* = 1, …, *n*) and each pollen *u* (*u* = 1, …, *v*, where *v* denotes the number of pollens), we computed the Spearman correlation coefficient *r*_*iu*_ by using the coefficients of their corresponding functional curves. The significance of the correlation coefficient was then evaluated. We assumed that patient *i* was allergic to pollen *u* if *r*_*iu*_ was significantly larger than 0. Taking into account that the functional coefficients are dependent [6], the usual correlation test could not be applied. For this reason, a permutation (or randomization) test [59–61] was considered. It performs a statistical significance test with weaker assumptions. The central one is that of exchangeability, allowing for relaxing the fundamental assumption of independence in the classical test theory. The number of permutations used for the correlation test was 10,000 [62]. Note that permutation tests are implemented in R in several packages such as, for instance, the package jmuOutlier [63].

Let *p*_*iu*_ be the *p*-value resulting from the permutation correlation test. If *p*_*iu*_ > α, where α is the significance level (in our study we used α = 0.05), we assumed that there was not statistical evidence that patient *i* was correlated with pollen *u* and, thus, we had *ARp* = 0. If *p*_*iu*_ ≤ α, the *ARp* index was computed as the ratio between *r*_*iu*_ and the sum of the correlations between the patient and all the pollens significantly larger than 0. Therefore, *ARp*_*iu*_, the *ARp* index for patient *i* and pollen *u* was equal to 0 if *p*_*iu*_ < 0.05 and to *r*_*iu*_ / ∑_*u*|*piu≤*α_ *r*_*iu*_, otherwise. By computing the *ARp* index for all the patients we obtained the results summarized in Table 7. Table 7 contains, for each cluster, the weighted means of *ARp* distinguished by cluster with weights given by the membership degrees. Note that, for each cluster, the mean values were computed by considering only the patients assigned to the cluster. In Table 8 we also report the percentages of times in which the patients assigned to a cluster had the *ARp* index equal to 1 for a pollen.

**Table 8:** Mean values of *ARp* and percentages of times in which *ARp* = 1 distinguished by cluster.

	Gramineae	Olea	Urticaceae
Cluster 1 (*n*_1_ = 27)	0.23 (19.2)	0.15 (7.7)	0.19 (15.4)
Cluster 2 (*n*_2_ = 23)	0.31 (19.0)	0.20 (9.5)	0.11 (4.8)
Cluster 3 (*n*_3_ = 21)	0.61 (55.6)	0.11 (5.6)	0.06 (5.6)
Total	0.36 (28.2)	0.16 (7.0)	0.15 (9.9)

From Table 8 we can see that the Gramineae was the most common allergenic. The dynamic of the CSMS values were highly correlated with the Gramineae counts. With respect to the Gramineae, 28.2% of patients were such that *ARp* = 1. This percentage remarkably increased for Cluster 3 (55.6%). Moreover, the mean value of *ARp* for Cluster 3 was the highest (0.61). For all of these reasons, such a cluster was interpreted as ‘‘Gramineae allergy”.

Concerning the stability of the obtained solution, we found that, apart for a few exceptions, the use of the cut-off equal to 25% did not modify the cluster assignments of the patients. This did not hold for the cut-off equal to 50% that was, therefore, too mild for the Ascoli Piceno data. Furthermore, by considering the most complex functional model with *m* = 6 and *q* = 20, the solution of F*k*MedFD setting *f* = 1.5 and *k* = 3 was virtually equal to the one previously interpreted.

Finally, we compared the F*k*MedFD solution with those of its competitors reported in Table 4. As for the Berlin case, we used default options and set the same parameters as for F*k*MedFD, i.e., *k* = 3 and, for fuzzy methods, *f* = 1.5. Moreover, the clustering methods for functional data were applied to the functionals obtained setting the same values of the parameters *m*, *q* and λ. Note that, with respect to funHDDC, no partition was found by using default options. Thus, we used the option init = “random” such that the algorithm was run 20 times and the solution maximizing the log-likelihood was kept. We did it four times in order to assess the stability of the obtained solutions, and found that three times the same solution was attained. Such a solution was used for comparison purposes.

**Fig 4:**
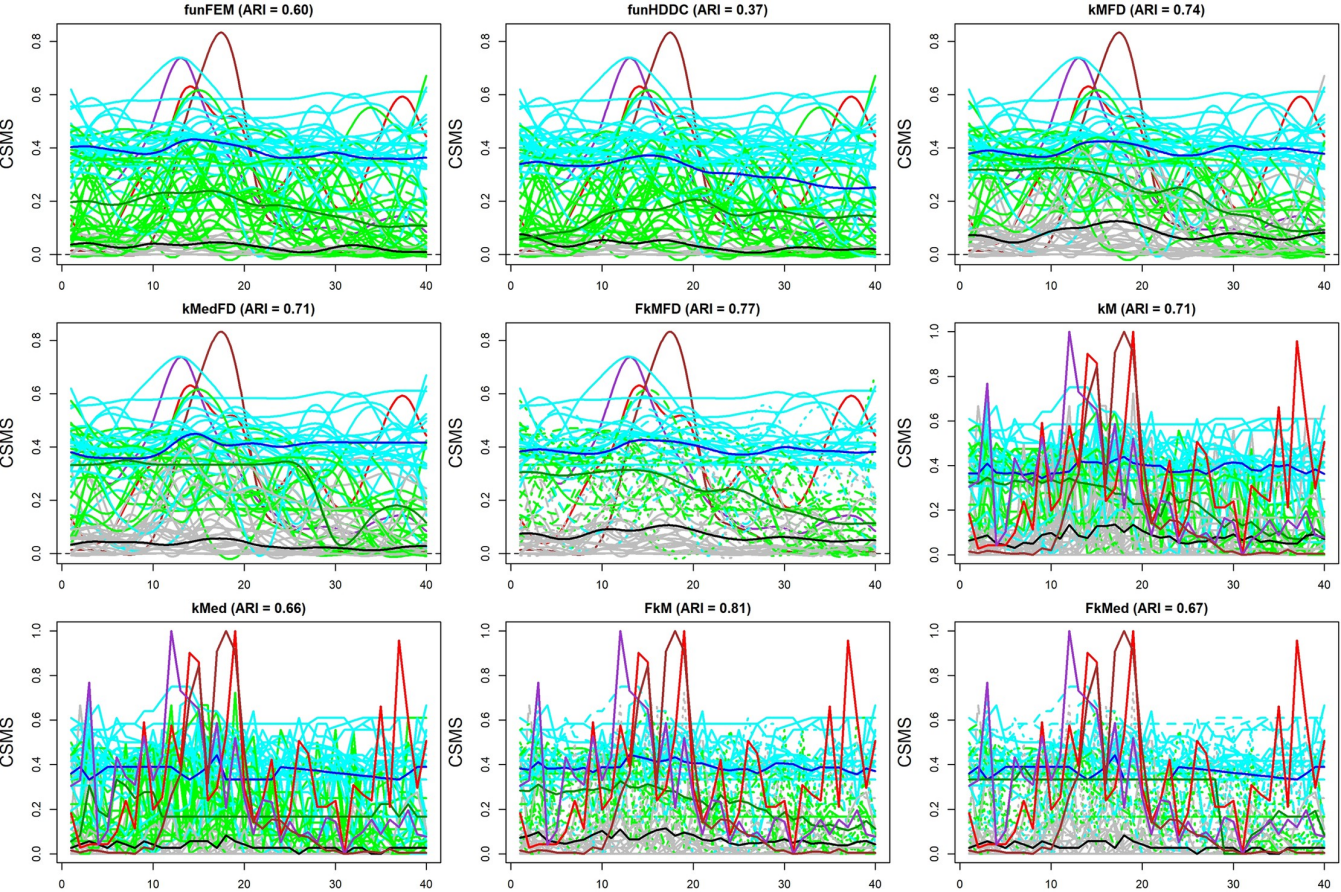
Plot of the solutions of the methods reported in Table 4 and of the pollens (red, violet and brown functionals). Cyan, grey and green functionals identify patients assigned to Cluster 1 (medoid in blue), Cluster 2 (medoid in black) and Cluster 3 (medoid in dark green), respectively. Solid, dashed and dotted functionals denote membership degrees higher than 0.90, between 0.70 and 0.90 and between 0.50 and 0.70, respectively.

The results, displayed in Fig 4, showed that the nine clustering methods identified rather different clusters with respect to the F*k*MedFD ones although, consistently with F*k*MedFD, all the competitors discovered clusters characterized by different levels of severity of the symptoms. The main difference among the partitions was related to Cluster 3 (green coloured curves). The cluster size and the medoid/centroid noticeably differed. The lowest ARI values were observed in connection with medoids/centroids with symptoms and drug intakes pretty stable during the reference time, thus in contrast with the features of the corresponding F*k*MedFD medoid. Once again, funFEM and funHDDC produced a hard partition with posterior probabilities equal to either 0 or 1, thus highlighting their tendency to discover hard partitions.

## Conclusion and future work

The paper focused on clustering for longitudinal mHealth data observed on a set of patients with the aim of opening the possibility of precision medicine. Given the large amount of noise in mHealth data, the suggestion is to convert mHealth data into functional data to denoise it. In order to discover clusters of homogenous patients, we proposed to apply the fuzzy *k*-medoids algorithm to the obtained functional coefficients (F*k*MedFD). By the B-spline basis system, these coefficients allow for finding continuous smoothing functions synthesizing the general trend of the observed data. The peculiarities of F*k*MedFD, are:

- The use of smoothing techniques in order to remove the noise of the recorded mHealth data;
- The adoption of the fuzzy approach to clustering that makes the method more flexible to handle all those situations with unclear assignments;
- The use of medoids to interpret and characterize the clusters: this is in general more natural than the use of centroids and appears particularly effective for mHealth data due to its robustness properties;
- The ease of implementation by using standard software tools, making the method also suitable for non-expert users.

FkMedFD has been applied in order to analyze two mHealth datasets referring to patients affected by Allergic Rhinoconjunctivitis (AR) living in Berlin (German) and Ascoli Piceno (Italy). The studies have allowed us to identify groups of patients with similar levels of disease allowing for tailoring of treatments. The clusters we found were interpreted by considering S1 File related to the patients and trends in air concentration of the supposed environmental causes of the patient diseases. The Berlin data set was quite small and two clusters, distinguishing two levels of AR severity, were found. For the Ascoli Piceno data, three clusters of patients were discovered. As for Berlin, two clusters distinguished the patients with respect to the levels of AR severity (high and low). The additional cluster detected some patients suffering from AR and allergic to the Gramineae. This result was discovered by comparing the curves of the patients and those of the pollens and developing a new index for assessing their relationships based on permutation correlation tests.

Our studies showed how the joint use of fuzzy clustering and functional data analysis can be fruitfully applied for the analysis of mHealth data. The obtained findings may stimulate further research on the topic with particular reference to the characterizations of the clusters with respect to external information. This is fundamental for precision medicine. In this paper, we have partially explored this point in terms of some demographic variables and the sensibilization to some pollens. However, this should be further investigated by studying whether the clusters are similar with respect to the clinical or biological phenotypes for some characteristics so that the patients belonging to the same cluster may represent a specific diagnostic sub-phenotype and be treated ad-hoc.

## Supporting information

S1 File (ZIP)
